# A versatile assay to determine bacterial and host factors contributing to opsonophagocytotic killing in hirudin-anticoagulated whole blood

**DOI:** 10.1038/srep42137

**Published:** 2017-02-08

**Authors:** Erika van der Maten, Marien I. de Jonge, Ronald de Groot, Michiel van der Flier, Jeroen D. Langereis

**Affiliations:** 1 Laboratory of Pediatric Infectious Diseases, Radboud Center for Infectious Diseases, Radboud university medical center, Nijmegen, The Netherlands; 2 Pediatric Infectious Diseases and Immunology, Department of Pediatrics, Radboud university medical center, Nijmegen, The Netherlands

## Abstract

Most bacteria entering the bloodstream will be eliminated through complement activation on the bacterial surface and opsonophagocytosis. However, when these protective innate immune systems do not work optimally, or when bacteria are equipped with immune evasion mechanisms that prevent killing, this can lead to serious infections such as bacteremia and meningitis, which is associated with high morbidity and mortality. In order to study the complement evasion mechanisms of bacteria and the capacity of human blood to opsonize and kill bacteria, we developed a versatile whole blood killing assay wherein both phagocyte function and complement activity can easily be monitored and modulated. In this assay we use a selective thrombin inhibitor hirudin to fully preserve complement activity of whole blood. This assay allows controlled analysis of the requirements for active complement by replacing or heat-inactivating plasma, phagocyte function and bacterial immune evasion mechanisms that contribute to survival in human blood.

Blood is normally sterile, but in cases when epithelial barriers are compromised and the immune system is not optimally equipped to fight pathogens, bacteria can be present in the blood, which is called bacteremia. Bacteria have evolved various mechanisms that prevent opsonophagocytosis, contributing to their ability to colonize their host, but also occasionally resulting in severe infections. Overall, Gram-positive bacteria are protected from complement-mediated lysis by the presence of a thick outer cell wall consisting of peptidoglycan, which prevents the bacterial membrane from lysis by the pore-forming membrane attack complex^1^. Conversely, Gram-negative bacteria, which are characterized by an outer membrane surrounding the bacterial cell wall, are vulnerable to complement-mediated killing due to assembly and insertion of the membrane attack complex on the bacterial surface^2^. Several bacterial species express a polysaccharide capsule, that protects them from recognition by opsonizing antibodies and in Gram-negative bacteria such as *Haemophilus influenzae* from insertion of the membrane attack complex^3^.

Besides a protective capsule, which can be found on both Gram-positive and Gram-negative bacteria, many invasive bacteria are able to hijack human complement regulatory proteins, thereby decreasing complement activation on their bacterial surface. For instance, *Streptococcus pneumoniae, H. influenzae, Escherichia coli* and *Neisseria meningitidis* are able to bind human factor H^4^,^5^,^6^,^7^, which decreases alternative complement activation and thereby reduces C3 opsonization.

In order to study the complement evasion mechanisms of bacteria, or the capacity of complement to opsonize and kill bacteria, most *in vitro* studies performed to date are using serum, plasma or baby rabbit complement containing active complement for complement opsonization. For opsonophagocytosis, isolated phagocytes or phagocyte-like cell lines such as HL-60 are used^8^,^9^,^10^,^11^. However, this is by no means representative to the real live situation in whole blood. For instance, the isolation of neutrophils leads to priming, which affects the ability of the neutrophils to form reactive oxygen species and changes their responses to cytokines^12^. In addition, serum has altered levels of coagulation proteins compared to plasma in whole blood. An example is plasminogen^13^, which can bind to the bacterial surface of *S. pneumoniae* and is involved in bacterial virulence^14^,^15^. Another example is fibrinogen, shown to bind to *Streptococcus pyogenes* M protein, which decreases C3b deposition and opsonophagocytosis^16^,^17^.

To circumvent these limitations in order to study complement-mediated opsonophagocytosis of bacteria, we explored the possibility to use whole blood directly after venous puncture for use in opsonophagocytosis assays. Here, we describe a versatile and easy to perform whole blood killing assay in which both phagocyte function and complement activity can be monitored and modulated. We used a selective thrombin inhibitor hirudin, which preserved complement activity of whole blood, in contrast to lithium heparin, sodium heparin, EDTA or sodium citrate.

## Material and Methods

### Ethics statement

After informed consent, a venous blood specimen was collected from the median cubital vein of healthy volunteers (age, 20–40 years; both males and females). Collection of blood was approved by the Ethics Committee of the Radboud University, Nijmegen, the Netherlands and experiments were carried out in accordance with local guidelines and regulations and complies with the Declaration of Helsinki and the Good Clinical Practice guidelines.

### Bacterial growth conditions

*Streptococcus pneumoniae* strain TIGR4^18^, *Streptococcus pneumoniae* strain TIGR4Δ*pspC*^19^, *Klebsiella pneumoniae* RUMC-KP01 (Clinical isolate Medical Microbiology, Radboud UMC Nijmegen, the Netherlands), *Staphylococcus aureus* strain NCTC 8178 (National Collection of Type Cultures), *Escherichia coli* BL21 DE3 (Agilent), *Neisseria meningitidis* serogroup B strain H44/67^20^, *Pseudomonas aeruginosa* ATCC15692 (American Type Culture Collection), *H. influenzae* type A strain ATCC 9006 (American Type Culture Collection), *H. influenzae* type B strain ATCC 10211 (American Type Culture Collection), non-typeable *H. influenzae* (NTHi) strain R2866^3^, NTHi strain 3655^21^ and NTHi strain 11P6H^22^ were used in this study. *H. influenzae* was grown under shaking conditions at 37 °C in brain heart infusion (BHI) broth (Becton Dickinson) supplemented with 10 μg/mL haemin (Sigma-Aldrich) μg/mL β-nicotinamide adenine dinucleotide (Merck) (sBHI). *S. pneumoniae* was grown under static conditions at 37 °C with 5% CO_2_ in Todd-Hewitt broth supplemented with 5 g/L yeast extract. *N. meningitidis* was grown on blood agar plates and collected directly from overnight plates. *K. pneumoniae, S. aureus, E. coli* and *P. aeruginosa* were grown under shaking conditions at 37 °C in Luria-Bertani (LB) broth.

### IgG, IgM and C3 opsonization assays

Blood for serum collection was collected in SST II *Advance* tubes (BD, Ref. 367953). Tubes were inverted after blood was drawn, incubated for 15 minutes at room temperature to clot, centrifuged with 3000 × g for 15 min at room temperature and serum was stored in small aliquots at −80 °C.

Blood for plasma preparation was collected in K2E (EDTA) tubes (BD ref. 367864), Trisodium citrate tubes (BD ref. 363047), Sodium heparin tubes (BD ref. 367869), Lithium heparin tubes (BD Ref. 368496) or S-Monovette r-Hirudin tubes (Sarstedt, ref. 04.1944.001). Tubes were inverted after blood was drawn, centrifuged with 3000 × *g* for 15 min at 4 °C and plasma was stored in small aliquots at −80 °C.

For human IgG, human IgM and human C3 binding, bacteria (1.10E7 in 100 μL) were incubated with 10% plasma or serum in Hank’s Balanced Salt Solution (HBSS) without phenol red containing Ca^2+^/Mg^2++^ 0.1% gelatin (HBSS3+) for 30 min at 37 °C. Bacteria were washed and incubated with 1:500 diluted FITC-labelled poly-clonal goat anti-human C3 (MP biomedicals), 1:100 diluted FITC-labelled Fc-specific goat anti-human IgG (Sigma-Aldrich) or 1:100 diluted FITC-labelled μ-chain-specific goat anti-human IgM (Sigma-Aldrich) in PBS with 2% BSA for 30 min at 4 °C. Bacteria were washed and fixed for 20 min with 2% paraformaldehyde. Bacteria were taken up in PBS for flow cytometry.

### Whole blood killing assay

After informed consent was obtained, a venous blood specimen was collected from the median cubital vein of healthy volunteers (age, 20–40 years; both males and females) into S-Monovette r-Hirudin tubes (Sarstedt). Blood was kept at room temperature on a roller bench until used.

For the whole blood killing assay, 100 μL of hirudin-anticoagulated blood was added per well in a 96-well plate. Bacterial suspensions in PBS, containing 1.10E5 colony forming units (CFU), were added in a maximum volume of 5 μL and immediately mixed with the blood. The 96-well plate was incubated for the indicated time at 37 °C under continuous shaking. The number of bacterial CFU was determined at start and after incubation by plating serial 10-fold dilutions. The percentage of bacteria that survived was calculated.

For plasma inactivation, 100 μL of hirudin-anticoagulated blood was added per well in a 96-well plate and centrifuged at 1000 × *g* for 5 min. Plasma was removed and heat-inactivated for 20 min at 56 °C. Blood cells were washed by adding 100 μL PBS and centrifuged with 1000 × g for 5 min. PBS was removed and heat-inactivated plasma was mixed with the pelleted cells and used for the killing assay. To examine the effect of plasma alone on bacterial clearance, 200 μL hirudin-anticoagulated blood was centrifuged 1 min at 16.000 × *g* and 100 μL plasma was used for the killing assay in the absence of blood cells. For 50%, 25% and 10% active plasma, 50 μL, 25 μL and 10 μL active plasma was mixed with 50 μL, 75 μL and 90 μL heat-inactivated plasma, respectively, and was mixed with the pelleted blood cells and used for the killing assay. For plasma replacement, 100 μL of hirudin-anticoagulated blood was added per well in a 96-well plate and centrifuged at 1000 × *g* for 5 min. Plasma was removed and cells were washed by adding 100 uL PBS and centrifuged at 1000 × *g* for 5 min. PBS was removed and pooled hirudin-anticoagulated plasma was mixed with the pelleted cells and used for the killing assay. For C6-depleted serum (CompTech) and C6-deficient patient serum^23^, serum was diluted in PBS to 10%. Reconstitution of C6 was performed by supplementing 6.4 μg/mL purified C6 (CompTech) in 10% serum because manufacturer’s product description states full reconstitution of serum was achieved with 64 μg/mL.

Inhibitor cytochalysin D (cyto D) (Sigma-Aldrich), anti-complement receptor 3 (CR3) subunit CD11b antibody clone 44a (α-CD11b) (Gift from Prof. Leo Koenderman), 4-hydroxytamoxifen (4-OHT) (Sigma-Aldrich), factor H (FH) (CompTech) or an equal volume of PBS were added to the hirudin-anticoagulated blood before adding the bacteria.

### Phagocytosis of CFSE-loaded *S. pneumoniae*

*S. pneumoniae* was grown in Todd-Hewitt broth supplemented with 5 g/L yeast extract to OD_620_ = 0.2, washed with PBS and labelled with carboxyfluorescein succinimidyl ester (CFSE) (Sigma-Aldrich) as previously described^24^. Five microliter (~1.10^6 ^CFU) CFSE-labelled bacteria were added to 100 uL hirudin-anticoagulated whole blood and incubated for 30 min. Red blood cells were lysed in ice-cold NH_4_Cl solution (8.3 g/L NH_4_Cl, 1 g/L KHCO_3_ and 37 mg/L EDTA) and washed once with ice-cold NH_4_Cl solution followed by a wash with PBS. Cells were stained with 1:200 diluted Alexa647-labelled α-CD16 (BD biosciences), 1:50 diluted V500-labelled α-CD3 (BD biosciences), 1:50 diluted PE-Cy7-labelled α-CD14 (Biolegend), 1:100 diluted BV421-labelled α-CD66b (BD biosciences) for 15 min. at room temperature. Cells were washed with PBS and analyzed by flow cytometry using a FACS LSR II (BD Biosciences). Data were analyzed using FlowJo v10.1.

## Results and Discussion

### Hirudin-anticoagulated blood is optimal for complement preservation

We used *Streptococcus pneumoniae* as model organism to set-up a whole blood killing assay because this bacterium is causing bacteremia in immune competent individuals^25^,^26^. In order to survive in blood, this bacterium has developed various mechanisms that inhibit recognition by the immune system^27^. For efficient opsonophagocytic killing, C3b opsonization of the bacterial surface of *S. pneumoniae* is required^28^. To determine which anticoagulants preserved complement C3b opsonization capacity, we determined IgG, IgM and C3 binding to the bacterial surface of *S. pneumoniae* after 30 minutes with 10% human serum or 10% human plasma anticoagulated with hirudin, lithium heparin, sodium heparin, EDTA or sodium citrate.

**Figure 1:**
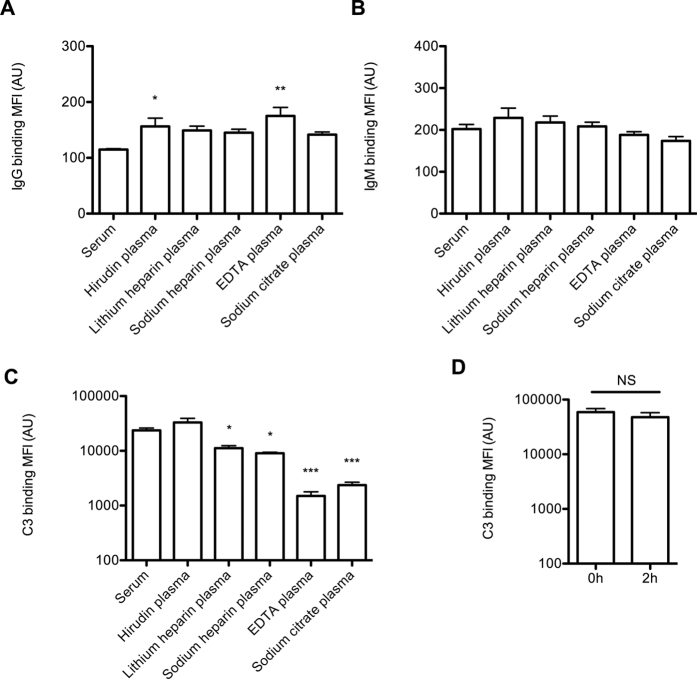
Plasma and serum IgG, IgM, C3 opsonization of *S. pneumoniae*. Bacteria (1.10E7) were incubated for 30 minutes in HBSS3+ containing 10% plasma anticoagulated with hirudin, lithium heparin, sodium heparin, EDTA or sodium citrate or serum from the same donor and binding of (**A**) IgG, (**B**) IgM, and (**C**) C3 was determined by flow cytometry (n = 3). One-way analysis of variance (ANOVA) with Dunnett’s Multiple Comparison Test was used for statistical analysis. *p < 0.05, **p < 0.01. (**D**) Hirudin anticoagulated blood was immediately (0 h) or after 2 hours rolling on a roller mixer (2 h) centrifuged and plasma was stored. Bacteria (1.10E7) were incubated for 30 minutes in HBSS3+ containing 10% of the plasma that was immediately or after 2 hours stored, and binding C3 was determined by flow cytometry (n = 3). A one-tailed student t-test was used for statistical analysis. NS = not significant.

Binding of IgG to the bacterial surface of *S. pneumoniae* incubated with 10% hirudin or EDTA anticoagulated human plasma was slightly increased compared to 10% human serum, whereas no significant differences for IgM were observed. More striking were the differences in C3 opsonization. Here, hirudin anticoagulated plasma showed the highest C3 opsonization of *S. pneumoniae*, whereas all other anticoagulants showed a significant decrease in C3 opsonization. Complement activity was preserved for at least 2 hours when blood was kept at room temperature (Fig. 1D). From these data, we conclude that hirudin anticoagulated plasma is superior in preserving complement activity.

Previously, Ison *et al*. determined killing of *Neisseria meningitidis* in citrate and heparin-anticoagulated whole blood^29^. In this study, heparin-anticoagulated whole blood was superior in killing *N. meningitidis* serogroup A compared to citrate-anticoagulated whole blood. In subsequent experiments, the same group compared this whole blood killing assay to serum bactericidal assay with blood from vaccinated children and consistently showed increased sensitivity for the whole blood killing assay^30^,^31^. Also, they showed a reduction of survival of *N. meningitidis* in the whole blood killing assay with increasing age of patients^32^. Whole blood killing of *N. meningitidis* has also been performed with hirudin-anticoagulated whole blood. Welsch *et al*. showed efficient killing of *N. meningitidis* serogroup B with whole blood from adults^33^. A slightly modified whole blood killing assay, with 25% heat-inactivated serum, showed increased killing with post-immunization serum compared to pre-immunization serum^34^. Comparisons in whole blood killing between huridin and other anticoagulants have not been studied previously.

The differences in complement activity preservation can largely be explained by the function of the different anticoagulants. Lithium heparin and sodium heparin induce a conformational change of antithrombin III to accelerate the inhibition of thrombin and factor Xa, thus preventing thrombin activation and the generation of fibrin. However, heparin is known to bind different proteins in the complement cascade^35^, as well as calcium and magnesium ions^36^, thereby affecting complement activity. Sodium citrate prevents blood from clotting through chelation of calcium ions by forming calcium citrate and EDTA scavenges bi-valent cations, such as calcium and magnesium, both are also required for complement activation. In contrast, hirudin (also known as lepirudin) is a highly specific thrombin inhibitor that does not interfere with complement activation^37^.

Hirudin has previously also been used in whole blood stimulation assays^33^,^38^,^39^,^40^. This enables to determine the contribution of cross-talk between complement and other factors such as cytokine release^38^, oxidative burst^40^ and phagocytosis^39^.

Even though thrombin is not directly involved in complement activation, there are some reports where it has shown to modulate complement activity. For instance, in C3−/− mice, thrombin was overexpressed and showed to cleave C5 into C5a and C5b^41^. In these studies, hirudin reduced acute lung inflammatory injury in C3−/− mice, but had no effect in C3+/+, indicating that thrombin-mediated cleavage of C5 only contributed to acute lung inflammatory injury when C3 is absent.

### Whole blood killing assay

Many bacterial pathogens such as *S. pneumoniae, Staphylococcus aureus, Klebsiella pneumoniae N. meningitidis* and *H. influenzae* frequently cause invasive disease, including sepsis^42^,^43^,^44^. When present in the blood, bacteria need to withstand the bactericidal activity of the complement system, and phagocytosis by peripheral blood neutrophils. We used hirudin anticoagulated blood to determine the survival of invasive bacterial pathogens in blood. For these experiments, we used *S. pneumoniae* strain TIGR4, originally isolated from the blood of a 30-year-old male^18^,^45^.

The whole blood killing assay is an easy-to-use opsonophagocytic assay to determine survival of bacterial pathogens in blood. Bacteria are added to 100 μL hirudin anticoagulated blood in a 96-wells round bottom plate and incubated at 37 °C while shaking to prevent sedimentation. Different inoculums (10^3^–10^5 ^CFU/100 μL blood) were tested and all showed a decrease in CFU counts over time (data not shown). For subsequent experiments, 10^5 ^CFU/100 μL blood were used.

**Figure 2:**
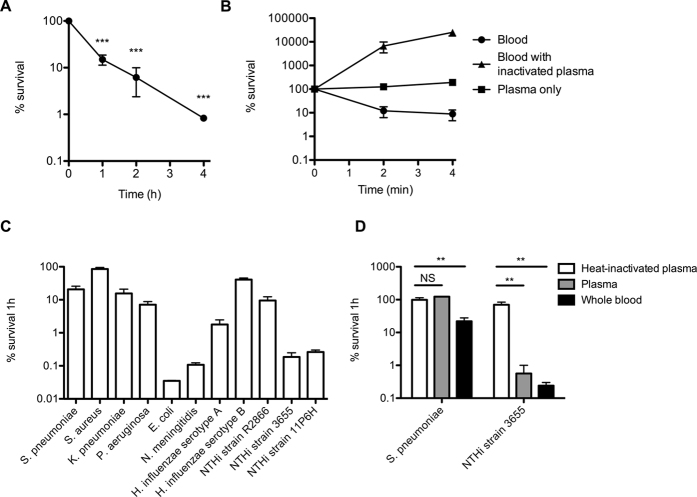
Phagocytes and active complement are required for efficient opsonophagocytic killing of *S. pneumoniae* in whole blood. (**A**) Bacterial survival in hirudin anticoagulated whole blood was determined after 1, 2 and 4 hours incubation (n = 3). One-way analysis of variance (ANOVA) with Dunnett’s Multiple Comparison Test was used for statistical analysis. ***p < 0.001. (**B**) Bacterial survival in whole blood, blood with heat-inactivated plasma and plasma only was determined after 1, 2 and 4 hours incubation (n = 7). (**C**) Killing in hirudin anticoagulated whole blood of *S. pneumoniae, S. aureus, K. pneumoniae, P. aeruginosa, E. coli, N meningitidis* and *H. influenzae* were determined after 1 hour incubation (n = 3). Killing of *S. pneumoniae* and non-typeable *H. influenzae influenzae* (NTHi) strain 3655 was determined with heat-inactivated (HI) hirudin plasma, hirudin plasma and hirudin anticoagulated whole blood after 1 hour incubation (n = 2).

We determined killing of *S. pneumoniae* in whole blood and observed significant killing already after 1 hour, which increased further in time (Fig. 2A). In order to determine the role of complement activity and phagocyte function, we performed the whole blood killing assay with either heat-inactivated plasma (see Material and Methods section for procedure) or with only plasma containing active complement. Whereas *S. pneumoniae* was killed in blood, no killing was observed with plasma only, indicating that phagocytes are required for efficient killing (Fig. 2B). When heat-inactivated plasma was mixed with blood cells, S. *pneumoniae* was able to grow very rapidly. This indicates that active complement is required for effective opsonophagocytosis as well, but also shows that whole blood contains sufficient nutrients for fastidious growth. The contribution of active complement in *S. pneumoniae* opsonophagocytosis is known for a long time^46^,^47^,^48^, and our results are consistent with these studies.

We determined whole blood killing after 1 hour for different pathogens that cause bacteremia^42^,^43^,^44^. Survival of *S. pneumoniae* strain TIGR4 was 20%. Similar survival was found for *K. pneumoniae* (16%) and *P. aeruginosa* (7%), whereas survival for *S. aureus* (85%) or *H. influenzae* serotype B (41%) were higher. Survival of *H. influenzae* serotype A (2%), *E. coli* (0.03%) and *N. meningitidis* (0.1%) was much lower. Survival of NTHi was strain dependent, 9% for R2886, but only 0.2% and 0.3% for strains 3655 and 11P6H, respectively. These strain-dependent differences in survival are probably due to variance in complement resistance since we have previously shown that survival in pooled human serum was much lower for NTHi strains 3655 and 11P6H as compared to strain R2866^4^.

In order to compare complement-mediated killing and opsonophagocytic-dependent killing for Gram negative and Gram positive bacteria, we determined survival of *S. pneumoniae* and NTHi strain 3655 with heat-inactivated plasma, plasma and whole blood. Both plasma and whole blood showed significant killing of NTHi strain 3655, whereas this was only the case with whole blood for *S. pneumoniae* (Fig. 2D). These data clearly indicate that killing of Gram negative, unencapsulated, NTHi strain 3655 was largely dependent on complement-mediated killing, whereas killing of *S. pneumoniae* was dependent on complement activation and opsonophagocytosis.

### Modulation of bacterial, cellular and humoral factors contributing to whole blood killing

**Figure 3:**
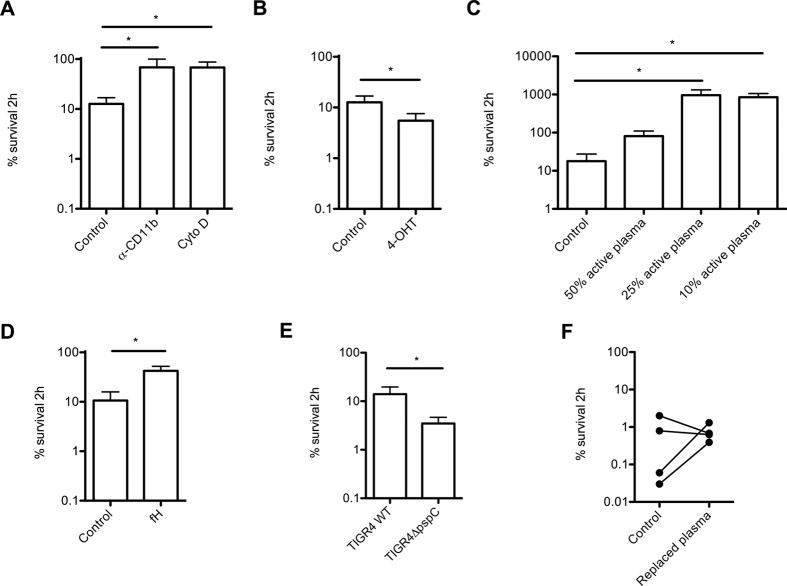
Modulation of *S. pneumoniae* killing by modulating phagocytosis or complement activity. Bacterial survival in hirudin anticoagulated whole blood was determined after 2 hours incubation in the presence of (**A**) 10 μg/mL CD11b blocking antibody (α-CD11b), 10 μM actin polymerization inhibitor cytochalysin D (CytoD), (**B**) 10 μM 4-hydroxitamoxifen (4-OHT) or (**D**) 100 μg/mL human factor H (fH). (**C**) Bacterial survival in hirudin anticoagulated whole blood and blood with 50%, 25% and 10% active plasma was determined after 2 hours incubation (n = 4). (**E**) Bacterial survival of TIGR4 wild-type (WT) and TIGR4Δ*pspC* were determined after 2 h in hirudin anticoagulated whole blood. (**F**) Bacterial survival of TIGR4 was determined after 2 h in hirudin anticoagulated whole blood with or without plasma replacement. One-way analysis of variance (ANOVA) with Dunnett’s Multiple Comparison Test was used for statistical analysis (**A**,**C**). A one-tailed student t-test was used for statistical analysis (**B**,**D**–**F**). *p < 0.05.

With this whole blood killing assay, bacterial factors as well as host cellular and humoral factors can be modulated to determine their contribution to opsonophagocytic killing. For instance, blocking complement receptor 3 (CR3) with α-CD11b antibody 44a decreased killing of *S. pneumoniae* (Fig. 3A), indicating that recognition of C3b on the bacterial surface by phagocytes contributes to killing. The contribution of the CR3 in opsonophagocytosis is *S. pneumoniae* by neutrophils and macrophages is widely investigating^49^,^50^,^51^, and our results are consistent with these studies.

In addition, treatment of blood with cytochalysin D, an inhibitor for actin polymerization, also decreased killing of *S. pneumoniae* (Fig. 3A), indicating that killing was dependent on phagocytosis.

Recently, Corriden *et al*. showed that tamoxifen augmented neutrophil-mediated killing of *S. aureus, E. coli* and *Pseudomonas aeruginosa* through enhancing several pro-inflammatory pathways in human neutrophils, including chemotaxis, phagocytosis and neutrophil extracellular trap (NET) formation^52^. Here, we show that adding 10 μM 4-hydroxytamoxifen significantly augmented killing of *S. pneumoniae* in whole blood (Fig. 3B).

Killing of *S. pneumoniae*, but also other pathogens, is affected by the presence of opsonizing antibodies and the overall complement activity. To determine the role of complement activity, we used whole blood of which plasma was removed by centrifugation and replaced with 50%, 25% or 10% plasma containing active complement (see Material and Methods section for procedure). Replacement of the total amount of active plasma with 50% active plasma clearly decreased the killing capacity, which was even more apparent for 25% and 10% active plasma (Fig. 3C), indicating that decreasing the level of active complement reduces the capacity to clear *S. pneumoniae* from blood in a dose dependent manner.

Previously, we have used the whole blood killing assay to assess the contribution of human factor H in controlling complement activity and killing of *S. pneumoniae* by replacing plasma with factor H-depleted serum and supplementation with different concentrations of purified human factor H. In this assay, we showed that increasing human factor H to blood increased survival of *S. pneumoniae*, whereas decreasing factor H levels increased killing^53^. Adding 100 μg/mL factor H to whole blood decreased killing of *S. pneumoniae* significantly (Fig. 3D), which is in accordance with the findings that higher factor H levels decreased bacterial killing^53^. Binding of factor H to the bacterial surface was shown to protect many bacteria from complement-mediated opsonization^4^,^5^,^7^,^54^,^55^,^56^,^57^,^58^. Pneumococcal surface protein C (PspC) of *S. pneumoniae* is known to bind human factor H. In order to determine the role for factor H binding in whole blood killing we determined survival of a Δ*pspC* mutant and found that this mutant, as expected, had a decreased survival in whole blood (Fig. 3E). Overall, this demonstrates several possibilities in studying functions of complement in bacterial clearance using the whole blood killing assay.

While performing our whole blood killing experiments, we observed large inter-patient differences in *S. pneumoniae* survival (% survival 0.03–2.00) (Fig. 3F). To determine whether these differences can mainly be attributed to differences in plasma content or phagocyte function, we used whole blood from which plasma was removed by centrifugation and replaced it with pooled plasma in which the concentrations of opsonizing antibodies and the complement activity are constant (see Material and Methods section for procedure). In this assay, killing of *S. pneumoniae* was more consistent (% survival 0.39–1.30), compared to survival in blood from the four single donors (% survival 0.03–2.00) (Fig. 3F). These data indicate that mainly differences in plasma components (opsonizing antibodies and complement activity) between these four donors attribute to the inter-donor variation in whole blood killing capacity. This approach can also be used to determine vaccine-induced protection. Previously, Welsch *et al*. showed that supplementation of whole blood with 25% heat-inactivated post-vaccination serum increased killing of *N. meningitidis* compared to pre-immunization serum^34^. This approach enables comparison of whole blood killing of pathogens with different serum samples in combination with a single fresh blood donor.

### Contribution of C6 in opsonophagocytic-mediated killing of *N. meningitidis*

**Figure 4:**
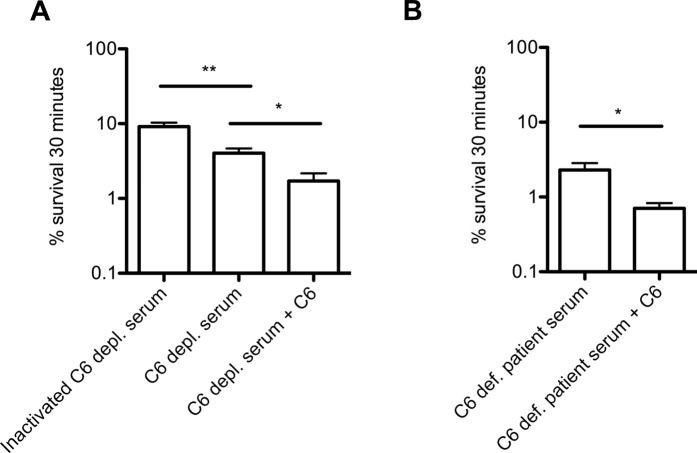
Contribution of C6 in opsonophagocytic-mediated killing of *N. meningitidis*. Bacterial survival of *N. meningitidis* strain H44/76 was determined after 30 minutes in hirudin anticoagulated whole blood with (**A**), 10% heat-inactivated serum, 10% C6-depleted serum, 10% C6-depleted serum supplemented with normal concentration C6 (see Material and Methods), (**B**) 10% C6-deficient patient serum, 10% C6-deficient patient serum supplemented with normal concentration C6. A one-tailed student t-test was used for statistical analysis. *p < 0.05, **p < 0.01.

Patients with deficiencies in the terminal complement components are more susceptible to invasive infections by *N. meningitidis*^59^. To mimic this in our whole blood killing assay, we replaced plasma with 10% C6-depleted serum and determined survival of *N. meningitidis* serogroup B strain H44/76 after 30 minutes. The presence of 10% heat-inactivated C6-depleted serum showed 9.1% survival (Fig. 4A), indicating that *N. meningitidis* serogroup B strain H44/76 is killed by complement-independent mechanisms, which has been described in literature previously. For instance, *N. meningitidis* serogroup C was killed by antibody-dependent cell-mediated antibacterial activity^60^ as well as opsonin-independent phagocytosis^61^. Opacity (Opa) proteins have been implicated to be important in opsonin-independent phagocytosis of *N. meningitidis*^62^,^63^ through neutrophil surface receptors CD66 and CR3^64^,^65^, whereas macrophages bind unopsonized *N. meningitidis* almost exclusively via the class A macrophage scavenger receptor^66^. Although not investigated in detail, we show that *N. meningitidis* serogroup B strain H44/76 is efficiently killed through complement-independent mechanism.

To study the contribution of complement in addition to complement-independent mechanisms, we used 10% C6-depleted serum in the whole blood killing assay. When 10% C6-depleted serum was used, survival was significantly lower (4.0%) compared to heat-inactivated serum (Fig. 4A). Since C6-depleted serum is not able to form a membrane attack complex, this increased killing is likely due to complement-dependent opsonophagocytosis. In order to restore terminal complex activity, we supplemented C6-depleted serum with C6 and observed a significant increase in killing, implicating that formation of membrane attack complex, next to opsonin-dependent and opsonin-independent killing, contributed to overall clearance of *N. meningitidis* from whole blood.

Previously, we have described a patient with a novel heterozygous missense mutation in the *C6* gene. Next to this novel heterozygous C6 mutation, a known heterozygous splice site variation was also identified, resulting in a C6 molecule that is 14% shorter due to a premature stop codon, but can still be build into the terminal complement complex, can kill bacteria, and is hemolytically active^67^,^68^. But, both mutations resulted in a lower (5%) C6 protein level. Normal immunoglobulin levels (IgG/IgA/IgM/IgE) and other complement factors (C3, C4) were found. When 10% C6-deficient patient serum was used, survival was low (2.3%) (Fig. 4B), which was consistent with results obtained with C6-depleted serum. Survival was significantly lower when C6 was reconstituted (0.7%) (Fig. 4B), indicating that C6 supplementation increased bacterial killing in whole blood.

Altogether, these results with C6-depleted serum and C6-deficient patient serum obtained similar results; decreased killing as compared to C6-reconstituted serum, which is consistent with the clinical phenotype of these patients.

### Monocytes and neutrophils contribute to opsonophagocytosis of *S. pneumoniae*

**Figure 5:**
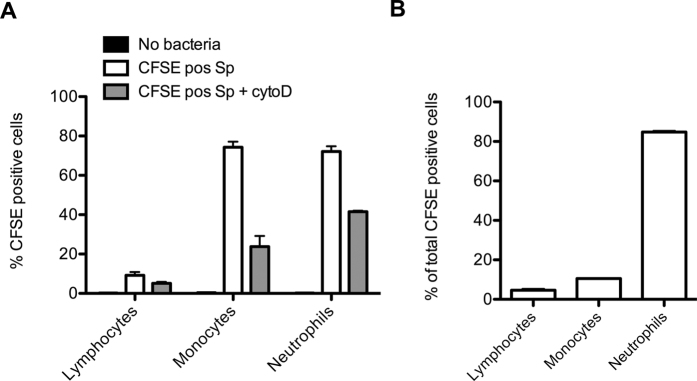
Whole blood killing of *S. pneumoniae* is mainly dependent on neutrophil-mediated opsonophagocytosis. (**A**) *S. pneumoniae* was loaded with 10 μM CFSE and incubated 30 min in hirudin anticoagulated whole blood. Erythrocytes were removed by hypotonic shock and the percentage CFSE positive lymphocytes (CD3 positive), monocytes (CD14 positive/CD16 negative) and neutrophils (CD16pos/CD66b positive) were determined by flow cytometry. (**B**) Percentage of CFSE positive cells were determined.

In order to address which cell type was predominantly responsible for opsonophagocytosis of *S. pneumoniae* in whole blood, we labelled *S. pneumoniae* with CFSE as previously described^24^. CFSE-labelled bacteria were added to whole blood in the absence or presence of cytochalysin D to block phagocytosis. Especially monocytes (74%) and neutrophils (72%) were found to bind and phagocytose *S. pneumoniae*, which was only 9% for lymphocytes (Fig. 5A). Cytochalysin D decreased *S. pneumoniae* association to monocytes, neutrophils and lymphocytes to 24%, 42% and 5%, respectively, indicating that half of the cells in the control condition actually phagocytosed *S. pneumoniae*, whereas the other half of the CFSE-labelled bacteria were cell-associated. When the total percentage of *S. pneumoniae* association with cells was determined, most of them, 85%, were neutrophils, 10% monocytes and 5% lymphocytes (Fig. 5B), indicating that neutrophils are the most important cell type for opsonophagocytosis of *S. pneumoniae* in whole blood. These results are consistent with previous literature where phagocytosis experiments showed efficient uptake of opsonized *S. pneumoniae* by both macrophages and neutrophils^69^,^70^,^71^. The important role for neutrophils in opsonophagocytic killing and protection against pneumococcal disease is supported by *in vivo* models wherein neutrophils were depleted^72^,^73^.

## Conclusion

The use of hirudin-anticoagulated whole blood enabled us to study the contribution of both bacterial and host factors in the killing of several pathogens, including *S. pneumoniae, K. pneumoniae, S. aureus* and *H. influenzae*. Complement activity preservation of hirudin was superior compared to lithium heparin, sodium heparin, EDTA or sodium citrate. Altogether, we describe a versatile assay to determine bacterial and host factors affecting opsonophagocytic killing of bacteria in hirudin-anticoagulated whole blood as a model for bacteremia.

## Additional Information

**How to cite this article:** van der Maten, E. *et al*. A versatile assay to determine bacterial and host factors contributing to opsonophagocytotic killing in hirudin-anticoagulated whole blood. *Sci. Rep.* **7**, 42137; doi: 10.1038/srep42137 (2017).

**Publisher's note:** Springer Nature remains neutral with regard to jurisdictional claims in published maps and institutional affiliations.
